# Olfactory identification deficits following Covid‐19 in older adults and its impact on cognition

**DOI:** 10.1002/alz70857_106381

**Published:** 2025-12-26

**Authors:** Latha Velayudhan, Maahek Kapasi, Ellie Pickering, Megan Richards, Abbie Palmer, Zunera Khan, Anne Corbett, Clive G Ballard, Dag Aarsland

**Affiliations:** ^1^ Institute of Psychiatry, Psychology and Neuroscience, King's College London, London, United Kingdom; ^2^ University of Exeter, Exeter, United Kingdom

## Abstract

**Background:**

The coronavirus disease 2019 (Covid‐19) is an ongoing viral pandemic and smell dysfunction is one of the symptoms. Olfactory impairment in general has been associated with increased mortality, poor quality of life, increased incidence of depression and risk for dementia. Smell dysfunction related to Covid‐19 in older adults and its impact is lesser studied. We tested olfactory identification deficits in older adults above 50 years of age with and without smell problems following COVID‐19 infection.

**Methods:**

A longitudinal online survey, Smell and Taste Function in Older Adults in the Covid‐19 Pandemic (STAP) was added as a sub‐study to the ongoing remote wider study‐ Platform for Research Online to investigate Genetics and Cognition in Ageing (www.protect.org.uk). A separate ethics approval was sought for STAP (Reference LRS‐19/20‐18549, 03.06.2020). Interested participants of PROTECT study within the UK completed online questionnaire following consent, on a quarterly basis for a year for information on Covid‐19 symptoms including smell and taste loss, self‐reported cognitive problems, testing, diagnosis, treatment, in addition to socio‐demographic details. University of Pennsylvania Smell Identification Test (UPSIT) kits were used to test object smell deficits in a subgroup.

**Results:**

Of the 1017 participants completed the survey 437 participants reported having had Covid‐19 infection. We selected 150 individuals with and without smell loss following Covid‐19 infection (*n* = 75 in each group, matched for age and gender) and posted out UPSIT kits for self‐testing. The mean age in years was 69.21 (± 6.55) with 80 women (53.3%) and total UPSIT score was 22.54 (±12.27). The 2 groups were not different in their total UPSIT scores (*p* = 0.555) however when compared for gender, women scored higher than men (*p* = 0.002). We will present further results related to association with cognition and other factors.

**Conclusions:**

Older adults with and without smell dysfunction following Covid‐19 infection had similar UPSIT scores when tested after 2 years. This is interesting as it appears there was no difference in at least smell identification function in the 2 groups. Further research is needed to understand the longitudinal course of smell impairment related to Covid‐19 in larger samples and risk for developing cognitive impairment.

